# Transcriptomic profiling in canine B-cell lymphoma supports a synergistic effect of BTK and PI3K inhibitors

**DOI:** 10.3389/fvets.2025.1577028

**Published:** 2025-04-25

**Authors:** Xenia Lainscsek, Weibo Kong, Barbara C. Rütgen, Julia Beck, Bertram Brenig, Ingo Nolte, Hugo Murua Escobar, Leila Taher

**Affiliations:** ^1^Institute of Biomedical Informatics, Graz University of Technology, Graz, Austria; ^2^Clinic for Hematology, Oncology and Palliative Care, Rostock University Medical Center, University of Rostock, Rostock, Germany; ^3^Department for Pathobiology, Clinical Pathology, University of Veterinary Medicine Vienna, Vienna, Austria; ^4^Chronix Biomedical GmbH, Göttingen, Germany; ^5^Institute of Veterinary Medicine, University of Göttingen, Göttingen, Germany; ^6^Small Animal Clinic, University of Veterinary Medicine Hannover, Hannover, Germany; ^7^Institute of Medical Genetics, Rostock University Medical Center, University of Rostock, Rostock, Germany; ^8^Institute for Biostatistics and Informatics in Medicine and Ageing Research, Rostock University Medical Center, University of Rostock, Rostock, Germany

**Keywords:** canine lymphoma, tyrosine kinase inhibitors, phosphoinositide 3-kinase inhibitors, RNA-seq, differential expression analysis, co-expression network analysis

## Abstract

**Introduction:**

B-cell receptor (BCR) signaling has revealed itself as a critical pathway in the pathogenesis of B-cell lymphoma. Within this pathway, the inhibition of Bruton's tyrosine kinase (BTK) or Phosphoinositide 3-kinases (PI3Ks) alone presents encouraging efficacy in the treatment of certain both canine and human hematological malignancies.

**Methods:**

Here we characterized the effects of the BTK inhibitor Ibrutinib and the PI3K inhibitor AS-605240 as single and combined agents in the canine pre-clinical diffuse large B cell lymphoma (DLBCL) model CLBL-1 by assaying cell proliferation and metabolic activity, and performing RNA-seq to measure gene expression changes.

**Results:**

We found 2,336 differentially expressed genes (DEGs) across all treatment types and time points relative to the control. The largest number of DEGs were induced by the combination of Ibrutinib and AS-605240. These genes were involved in adaptive immune response, leukotriene D4 metabolic and terms related to regulation of GTP and GTPase mediated signal transduction. Weighted gene co-expression network analysis (WGCNA) detected nine gene modules, five of which were associated with treatment response. Eighteen-percent of genes within these modules were also differentially expressed. Notably, we observed one module that was exclusively associated with the combined treatment whose gene members were related to cellular metabolism, homeostasis signaling, and protein synthesis and regulation.

**Conclusion:**

Narrowing in on highly connected genes of modules associated with treatment response with large fold changes across treatments which play roles in the main targeted pathways identified *PAG1, PRKAR2A, ACACA, FOS*, and *PRKCA* as potential primary candidates of the synergistic treatment effect.

## 1 Introduction

Canine lymphoma is a frequent and spontaneous cancer characterized by uncontrolled lymphocyte replication. Its most prevalent form is B-cell lymphoma, which accounts for 65–80% of all cases (1). Conventional first-line therapeutic intervention for canine lymphoma is L-CHOP (L-asparaginase, cyclophosphamide, hydroxydaunorubicin, vincristine, prednisolone) chemotherapy, which induces remission in nearly 85–90% of patients. However, the majority of patients develop resistance and relapse within the first 12 months after treatment, ultimately dying from their disease (2, 3).

In the last decade, tyrosine kinase inhibitors (TKIs) have emerged as promising therapeutic options for various cancers, including canine lymphoma (4, 5). TKIs are small molecules that compete with the adenosine triphosphate-binding site of tyrosine kinases, preventing their phosphorylation and activation, thereby inhibiting downstream signaling pathways (6). While some TKIs exhibit high specificity, most of them inhibit a broad range of kinases and impact multiple cellular processes (6). Specifically, TKIs targeting Bruton's tyrosine kinase (BTK) have demonstrated effectiveness in lymphoma treatment (5, 7). This enzyme plays a crucial role in B-cell receptor (BCR) signaling, mediating downstream events that regulate B-cell activation, proliferation, and survival. Specifically, antigen binding induces the aggregation of the BCR transmembrane protein with its co-receptors CD79a Molecule (CD79A) and CD79b Molecule (CD79B). This recruits and activates the members of the Src family of tyrosine kinases LYN (LYN Proto-Oncogene, Src Family Tyrosine Kinase), and SYK (Spleen Associated Tyrosine Kinase). Activated LYN and SYK phosphorylate the tyrosine residues located in CD79A and CD79B, in turn recruiting several other kinases and adaptor proteins that amplify the signal (8) and ultimately lead to the activation of phosphoinositide 3-kinases (PI3Ks). PI3Ks are a family of enzymes that convert phosphatidylinositol-4, 5-bisphosphate (PIP2)—a minor component of the plasma membrane—to phosphatidylinositol (3–5)-trisphosphate (PIP3) (9). PIP3 is a docking site for the members of the protein kinase B (AKT) family, PDK1 (Pyruvate Dehydrogenase Kinase 1) and BTK (10). Upon binding PIP3, AKT proteins undergo a conformational change, facilitating PDK1-dependent phosphorylation (11). Phosphorylated AKT proteins dissociate from the membrane and promote cell survival, proliferation, and growth through the phosphorylation and inhibition of several substrates, including FOXO transcription factors, the negative regulator of glucose homeostasis glycogen synthase kinase-3 beta (GSK3B), and the mTOR inhibitors TSC1 (TSC Complex Subunit 1) and TSC2 (TSC Complex Subunit 2) (11). Similarly, the binding of BTK to PIP3 facilitates its activation by upstream signaling molecules. Activated BTK activates PLCG2 (Phospholipase C Gamma 2) which hydrolyzes phosphatidylinositol 4,5-bisphosphate (PIP2) into diacylglycerol (DAG) and inositol trisphosphate (IP3) (12). DAG activates the members of the protein kinase C (PKC) family, a pivotal effector in the BCR signaling pathway that promotes NF-κB activation (13). Specifically, IκB kinase complex (IKK) activation by PRKCB (Protein Kinase C Beta) leads to the phosphorylation and degradation of the inhibitor of nuclear factor-κB (IκB) proteins, enabling nuclear factor kB (NF-κB) proteins to translocate to the nucleus, where they induce the transcription of genes needed for B-cell proliferation and survival (13).

Ibrutinib is a TKI that irreversibly blocks the activity of BTK by binding to its SH1/TK domain and can also prevent BTK autophosphorylation and thereby activation (14–16). A pioneer among BTK inhibitors, Ibrutinib has substantially improved outcomes in human lymphoma patients (17) and shows promise as a new frontier in canine lymphoma therapy (16, 18). Despite its effectiveness, the use of Ibrutinib is associated with several side effects, ranging from diarrhea, nausea, and fatigue to more severe complications such as cardiomyopathies and acute tumor lysis syndrome (19, 20). Furthermore, most patients treated with Ibrutinib eventually become refractory to therapy (19). The limitations of Ibrutinib have motivated the investigation of combination regimens.

Beyond BCR signaling, PI3Ks can be activated by multiple upstream pathways that couple a wide range of receptors with specific PI3K isoforms (21). Indeed, PI3K overactivity is a hallmark of cancer and not just B-cell malignancies. The pivotal role of PI3Ks in cancer has led to their investigation as promising therapeutic targets, but their toxicity profile has limited the ability of clinical trials to assess optimal dosage (21). AS-605240 is a strong and selective PI3K inhibitor (22). This compound prevents activation PI3K—specifically, phosphatidylinositol 4,5-bisphosphate 3-kinase catalytic subunit gamma isoform (PIK3CG)—by competitively binding to its ATP-binding site, thereby leading to reduced cell growth and proliferation, increase of apoptosis, and changes in cell migration and invasion (22, 23). PIK3CG forms mutually exclusive dimers with one of the regulatory subunits PIK3R5 and PIK3R6 (24). In addition, AS-605240 has been found to suppress phosphorylation of AKT proteins in cell lines with perturbed Ras expression (25). When activated, Ras facilitates the recruitment of PI3K—primarily, PIK3CG—to the cell membrane (26), thereby initiating the PI3K signaling cascade. Importantly, AS-605240 has demonstrated therapeutic potential in several disease models involving the PI3K pathway, including a murine model of cystic fibrosis (27).

In light of this, we recently characterized the effects of Ibrutinib and AS-605240 on cell proliferation and apoptosis in canine and human pre-clinical diffuse large B-cell lymphoma (DLBCL) models, and found that they can act synergistically (18). DLBCL is the most common lymphoma in humans, accounting for about 25–30% of all non-Hodgkin lymphoma (28, 29). Although it has a rapidly progressive course, it responds well to chemotherapy, and up to 50% of the patients achieve complete remission following chemotherapy (28, 29). In contrast, only 20% of canine patients with DLBCL survive 2 years after diagnosis despite therapy, having an average survival time of around 12 months (29). Parallels in molecular biology and therapeutic and patient management protocols make canine DLBCL a highly relevant spontaneous model for human DLBCL (30).

The aim of this study was to characterize the molecular mechanisms and identify the target genes that mediate the synergistic effects observed upon combined inhibition of the BTK and PI3K pathways by Ibrutinib and AS-605240, respectively, in a canine lymphoma model. For this purpose, we performed RNA-sequencing (RNA-seq) on CLBL-1, a canine B-cell line derived from a spontaneously occurring diffuse large B-Cell lymphoma (DLCL), upon single and combined treatment with Ibrutinib and AS-605240. We identified a total of 2,336 differentially expressed genes associated with biological processes relevant to lymphoma pathogenesis, including immune responses, lymphocyte proliferation, and apoptosis. Notably, 1,460 of these genes were exclusively differentially expressed upon the combined treatment with Ibrutinib and AS-605240, confirming their synergistic effect at molecular level. To gain deeper insights into the gene regulatory networks underlying this interaction, we then performed weighted gene co-expression network analysis [WGCNA (31, 32)]. This approach revealed a specific gene module exclusively associated with the combined treatment, with genes implicated in cellular metabolism, protein translation, and chromatin organization. Finally, thorough examination of the findings from integrated bioinformatics analyses within the context of the BTK and PI3K signaling pathways, revealed five potential primary gene candidates as targets of the synergistic treatment effect: *PAG1, PRKAR2A, ACACA, FOS*, and *PRKCA*.

## 2 Materials and methods

### 2.1 Cell line and culture condition

CLBL-1, the canine DLBCL cell line, was kindly provided by the developer of this cell line from the University of Veterinary Medicine in Vienna, Austria (33). CLBL-1 was cultured in a cell flask filled with RPMI-1640 medium, containing 10% heat-inactivated fetal bovine serum, and 1% penicillin-streptomycin (10,000 U/mL penicillin, 10 mg/mL streptomycin, Biochrom GmbH, Berlin, Germany). The cell flasks were set in an incubator at 37°C in a humidified atmosphere containing 5% CO_2_. The culture medium was replaced twice a week.

### 2.2 Kinase inhibitors

Two tyrosine kinase inhibitors, Ibrutinib (Ibr) and AS-605240 (AS) were purchased from Selleck Chemicals (Absource Diagnostics GmbH, Munich, Germany). According to the inhibitor handling instructions of the products, the powders of these inhibitors were dissolved in dimethyl sulfoxide (DMSO, Sigma-Aldrich Chemie GmbH, Steinheim, Germany) to prepare a stock solution with an initial concentration of 10 mM and stored at −80°C.

### 2.3 Inhibitor application experiment

CLBL-1 was seeded at the density of 3.3 × 10^5^ cells, separately incubated with serial end-concentrations of 1 μM Ibrutinib, 5 μM AS-605240 and their combination for different time periods. The concentrations for Ibrutinib and AS-605240 were chosen according to our previous study (18). Cells in the control group were cultured in the medium containing the same concentration of DMSO as the combined drug treatment group, i.e., 0.15% (v/v) the volume ratio of the inhibitor to total solution. When each respective time point was reached, incubated cells were harvested separately in order to assess the effects of inhibitors on cell proliferation, metabolic activity, and for RNA sequencing.

### 2.4 Trypan blue exclusion assay of cell viability

A total volume of 1.5 mL medium containing 5 × 10^5^ cells was seeded into each well of the 24-well plates, then exposed to the corresponding inhibitor or DMSO, two wells per treatment. When reaching 72 h, the cells were harvested and washed with PBS. The number of viable cells was determined by trypan blue dye exclusion (Marienfeld Superior, Lauda-Königshofen, Germany).

### 2.5 WST-1 cell proliferation/metabolic activity assay

A total volume of 150 μL medium containing 5 × 10^4^ cells was seeded into each well of the 96-well plates, then exposed to the corresponding inhibitor or DMSO, three wells per treatment. When reaching 72 h, 15 μL of pre-warmed WST-1 reagent was added into the cell suspension and medium background control, then incubated at 37° C for 2 h. Afterwards, the absorbance values of the samples against background control at 450 nm was detected by PromegaGloMax^®^-Multi Microplate Multimode Reader (Promega, Madison, WI, USA), and 750 nm was used as the reference wavelength.

### 2.6 Statistical analysis of cell proliferation and metabolic activity

The experiments of cell proliferation and metabolic activity were independently repeated at least four times. The significant difference between the DMSO, single-inhibitor, and combined-inhibitor exposure groups was evaluated in pairs using Tukey's multiple comparisons test of ordinary one-way ANOVA (34) (GraphPad Software, San Diego, USA). *P*-*values* ≤ 0.05 were considered to be significant.

### 2.7 RNA isolation for RNA-seq

A total volume of 6 mL medium containing 2 × 10^6^ cells was seeded into each well of 6-well plates, and then exposed to 1 μM Ibrutinib, 5 μM AS-605240, their combination, or DMSO, two wells per treatment. When reaching 24, 48, and 72 h respectively, incubated cells were harvested and washed three times with cool PBS and centrifuged at 200 × g for 8 min at 4°C. QIAzol^®^ Lysis Reagent (QIAGEN, Venlo, Netherlands) of 700 μL was added into the pellet of 5 × 10^6^ cells to lyse. Total RNA was isolated using the miRNeasy Mini Kit (QIAGEN, Venlo, Netherlands) referring to the manufacturer's Quick-Start Protocol. Genomic DNA contamination was prevented through the RNase-free DNase (QIAGEN, Venlo, Netherlands) digestion on the RNeasy^®^ Mini column. Finally, the concentration and quality of RNA in each sample was evaluated by Nanodrop Spectrophotometer ND1000 (PEQLAB Biotechnologie GmbH, Erlangen, Germany). The experiment was performed in triplicate, each replicate using CLBL-1 cells at a distinct passage number (121, 126, and 128).

### 2.8 RNA library construction and sequencing

Samples were prepared and sequenced in triplicates (Supplementary Table 1). A total RNA of 2 μg (with RNA integrity number >8) was used to prepare the sequencing libraries. Referring to the manufacturer's instruction, poly-A RNA was enriched and ligated to sequencing adapters with the NEBNext Ultra II RNA Library Prep Kit (New England Biolabs, Ipswich, USA). Single-end sequencing was conducted on IlluminaNextSeq500 sequencing platform (Illumina, San Diego, USA) to generate 75 bp-long reads. Raw data were deposited in the Gene Expression Omnibus (GEO) repository under the accession number GSE287971.

### 2.9 RNA-seq data preprocessing

Quality control was performed on raw sequencing data with FastQC [v0.11.9 (35)] and revealed no outliers among the samples. For each sample, reads from four lanes were pooled. Reads were mapped to the soft masked *Canine lupus familiaris* reference genome assembly CamFam3.1 (36, 37) and transcript quantification were simultaneously done with STAR [v2.7.5a (38)] using options “–quantMode GeneCounts” according to gene annotation from Ensembl (https://ftp.ensembl.org/pub/release-101/gtf/canis_lupus_familiaris/Canis_lupus_familiaris.CanFam3.1.101.chr.gtf.gz, last accessed in June 2024). The resulting bam files were indexed and sorted with SAMtools [v1.10 (39)] and mapping quality was assessed with qualimap [v2.2.a (39, 40)].

Genes associated with two or more reads across all samples of interest were considered expressed.

### 2.10 RNA-seq data analysis

#### 2.10.1 Gene expression values

Gene expression values were extracted from the “DESeq2” [v1.38.3 (41)] object derived from DESeqDataSetFromMatrix() (“design = ~1”) with the assay() function. Regularized-logarithm transformation was applied to the resulting matrix with the rlog() function.

#### 2.10.2 RNA-seq data clustering

Samples were clustered with the complete linkage method based on the expression value of each gene divided by its transcript length, using R's pheatmap() (42) function with default parameters. Dimensionality reduction was achieved with principal component analysis (PCA) to further verify sample clusters.

#### 2.10.3 RNA-seq data variance analysis

We evaluated sources of variation in gene expression across all samples based on linear regression models, using the R/Bioconductor package “variancePartition” (v1.32.5) (43, 44). Treatment type, incubation time, and cell passage number were assumed to be “fixed effects” and used as predictor variables for the expression value of each gene over each treatment, time point, and passage.

#### 2.10.4 Differential expression analysis

Differential expression analysis (DEA) was performed on expressed protein-coding genes using the DESeq() function of the “DESeq2” R package (v1.38.3) (41). Genes with a false discovery rate (FDR)-adjusted *P-value* ≤ 0.05 and an absolute log_2_ fold-change (log_2_ FC) >1 were considered differentially expressed.

On samples incubated with Ibr, AS, and IbrAS for 24, 48, and 72 h the analysis was performed using DMSO at the respective incubation times as control, adjusting for passage number, incubation time, and treatment (“design = ~ passage number + treatment and incubation time”). On samples of cells incubated with DMSO-only the analysis was done for 48 and 72 h using the 24 h sample as a reference, adjusting for passage number and incubation time (“design = ~ passage number + incubation time”).

#### 2.10.5 Principal component analysis (PCA) of differentially expressed genes

PCA was performed on the expression values of the differentially expressed genes using R's prcomp() (45) function.

#### 2.10.6 Weighted gene co-expression network analysis (WGCNA)

The top two-thirds of the expressed genes with highest expression values (10,138 genes) were considered for generating scale-free modules of co-expressed genes using the “WGCNA” R package (v1.72-5) (31, 32). The soft threshold power β was determined by calling the *pickSoftThreshold()* function on the matrix of gene expression values. β = 21 was the lowest power retaining approximate scale-free topology for a signed network (Supplementary Figure 1). A weighted adjacency matrix was generated from the gene expression matrix using the *adjacency()* function. The *TOMsimilarity()* function was used to compute the topological overlap matrix, which assesses the overlap of the neighborhoods of each pair of genes in the adjacency matrix and uses hierarchical clustering to group genes into “modules” based on their TOM-derived distances. Modules whose eigengenes had correlations >0.3 were merged to produce the final set of modules.

Associations between modules and treatments and/or incubation time were drawn from the linear regression models of eigengenes with treatment types or incubation times.

#### 2.10.7 Functional enrichment analysis

Functional enrichment analysis was performed with the enrichGO() function in the “clusterProfiler” R package (46). Only terms in the Gene Ontology (GO) category “biological processes” and pathways in the Kyoto Encyclopedia of Genes and Genomes (KEGG) were considered. Due to the fact that annotation for the canine genome is limited, we performed the analyses on the human orthologs of the canine genes. Orthologs and Entrez Gene IDs were retrieved from the BioMart database using the useMart() and getBM() functions in the “biomaRt” R package (v.2.54.0) (47, 48). The “host” parameter was set to “https://aug2020.archive.ensembl.org/.” Terms with at least five annotated genes in the test list and a false discovery rate (FDR)-adjusted *P-value* ≤ 0.05 were considered enriched.

#### 2.10.8 Hub genes

Hubs were defined as the top 5% genes with the highest “degree” (i.e., sum of connection weights of a gene to all other genes in the module, computed with the intramodular Connectivity() function of the WGCNA package) that have a “module membership” (i.e., correlation of a gene to its module eigengene, computed with the signedKME() function of the WGCNA package) of at least 0.8. Top hubs were defined as the genes with the highest intramodular connectivity (*kWithin*) and were obtained with the choose TopHubInEachModule() function.

The networks of the top 25 hubs in each module were represented using Cytoscape (49) with the forced-directed layout proposed by Kamada and Kawai (50) using the R plugin RCy3 (51). Parameters were set to a spring force constant (m_nodeDistanceStrengthConstant) of 15, a global scaling factor (m_nodeDistanceRestLengthConstant) 200, and an anti-collision strength (m_anticollisionSpringStrength) of 1.

#### 2.10.9 Motif discovery

*De novo* motif discovery within the promoter (±500 bp from the transcription start site) of the genes in relevant modules and also differentially expressed was performed with XSTREME (52) from the MEME suite (v5.4.0) (53). Matches to motifs in the JASPAR CORE database (release 2024) (54) were used to identify the transcription factors putatively recognizing the motifs. Instances of the motifs within the promoters were located with FIMO (55), also from the MEME suite.

Motif logos were visualized using the view_motif() function of the “universalmotif” (version 1.20.0) (56) R package.

#### 2.10.10 Pathway based data integration and visualization

The “pathview” (v1.30.1) (57) R package was used to map log_2_ FCs onto the graphs of the KEGG (58, 59) pathways “*B cell receptor signaling pathway*” and “*PI3K-Akt signaling pathway*.” Specifically, we first identified the human orthologs of the canine genes that were differentially expressed or members of potentially relevant WGCNA modules. We then mapped the log_2_ FCs of the corresponding canine genes onto the KEGG pathways “hsa04662” and “hsa04151.” Entrez Gene IDs were used for this purpose. This approach allowed us to leverage the extensive annotation of human pathways to gain insights into the functional implications of gene expression changes in canine lymphoma. In particular, BTK occurs in “hsa04662” but not in “cfa04662.”

## 3 Results

### 3.1 Ibrutinib combined with AS-605240 amplifies anti-proliferative and anti-metabolic effects on CLBL-1

**Figure 1:**
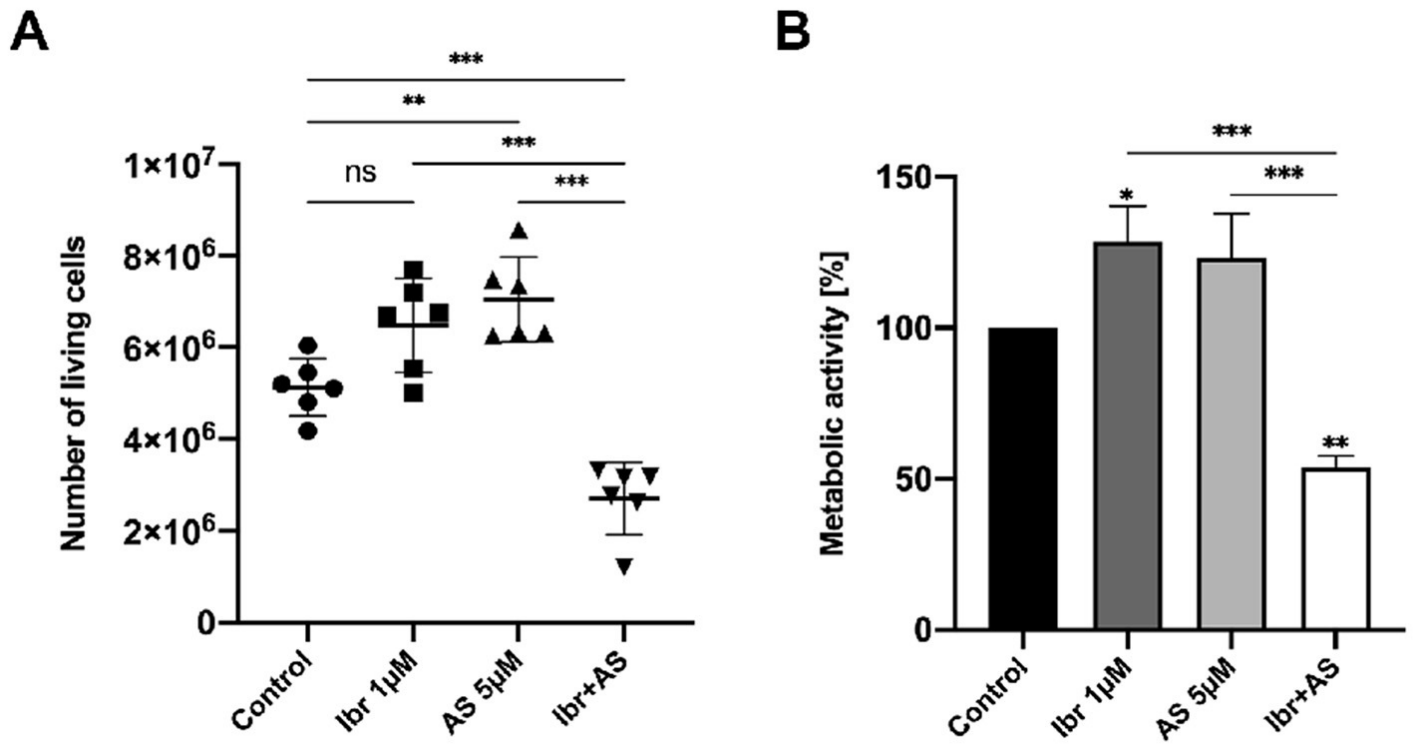
Combined application of Ibrutinib and AS-605240 for 72 h synergistically inhibits the proliferation and metabolic activity of CLBL-1. **(A)** Number of viable cells represented as dot plots. Means ± standard deviation (SD) of six independent experiments are shown. **(B)** Mean metabolic activity (%) compared to cells incubated with DMSO only, over three independent experiments. Error bars indicate one SD. Asterisks indicate statistical significance by a one-way Anova analysis with Tukey's multiple comparison test correction, with one, two or three asterisks if the *P*-*value* is below 0.05, 0.01, or 0.001, respectively.

To investigate the impact of combined BTK and PIK3CG inhibition on CLBL-1 cells, we assessed their proliferation and metabolic activity upon treatment with 1 μM Ibrutinib (Ibr), 5 μM AS-605240 (AS), and the combination of 1 μM Ibrutinib and 5 μM AS-605240 (Ibr + AS). Ibr and AS were dissolved in DMSO and therefore, cells treated with DMSO-only were used as a control group. Proliferation was evaluated with the Trypan blue exclusion assay and metabolic activity with the WST-1 proliferation assay. Compared to the number of viable cells of 5.1 million in the control group, 6.5 million in the Ibr group, and 7.0 million in the AS group, the combination of Ibrutinib and AS-605240 reduced the number of viable CLBL-1 cells to 2.7 million (*P-value* < 0.001, Figure 1A). In addition, compared to the percentage of metabolic activity of 100.0% in the control group, 128.4% in the Ibr group, and 123.1% in the AS group, metabolic activity of CLBL-1 was reduced to 53.8% in the Ibr+AS group (*P-value* < 0.01, Figure 1B).

### 3.2 Incubation with DMSO modulates gene expression changes driven by Ibrutinib and AS-605240

To elucidate the molecular mechanisms responsible for the observed amplification of anti-proliferative and anti-metabolic effects, we performed RNA-seq on CLBL-1 cells incubated with 1 μM Ibrutinib (Ibr), 5 μM AS-605240 (AS), 1 μM Ibrutinib, and 5 μM AS-605240 (Ibr + AS), or DMSO-only for 24, 48, and 72 h. Of the 19,947 protein-coding genes annotated in the canine reference genome assembly, about 76% (15,237) were considered expressed and retained for further analysis. Both hierarchical clustering of the Euclidean distance matrix and PCA of the gene expression profiles revealed one of the samples treated with Ibr for 72 h to be an outlier (Supplementary Figures 2A, B); this sample was consequently discarded in subsequent investigations. As anticipated, treatment type had the strongest effect on gene expression variation (30%), followed by the incubation time (10%), and lastly the cell passage number (5%) (Section 2; Supplementary Figure 2C).

**Figure 2:**
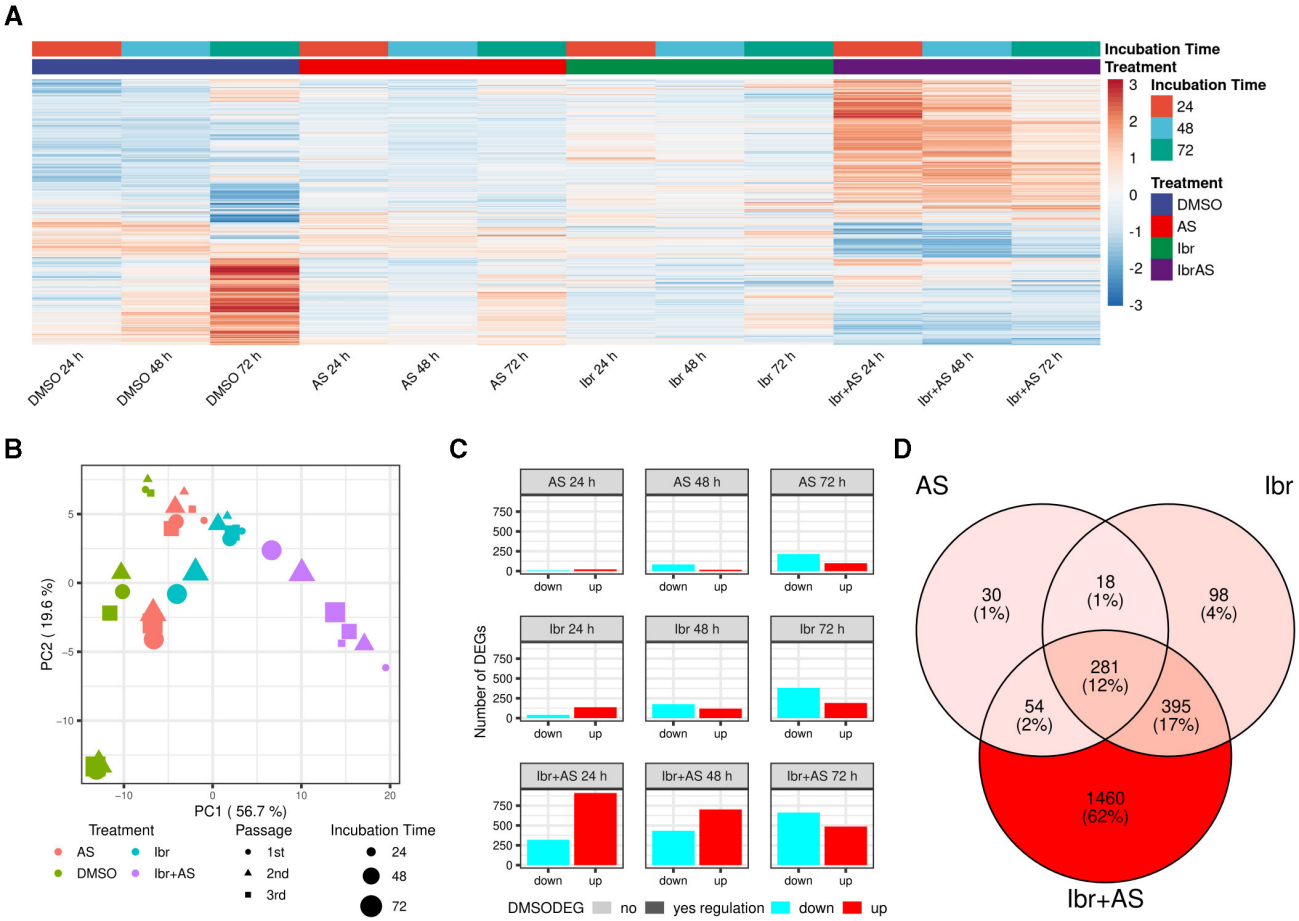
Ibrutinib and AS-605240 synergistically induce gene expression changes in CLBL-1. **(A)** Expression values of all 2,336 DEGs across treatments and incubation times. Expression values were scaled across all sample groups to a mean of zero and a standard deviation of 1 for each gene. Genes were hierarchically clustered with complete linkage based on the Euclidean distance of their expression profiles. Most lowly and highly expressed genes are evidently present in the combined Ibr + AS treatment groups across all time points and upon incubation with DMSO for 72 h, whereby a discordant trend is visible. **(B)** PCA plot showing how samples clustered based on the expression values of their DEGs. Samples cluster by treatment type and then by incubation time (57% and 20% variance explained, respectively). **(C)** Bar plot showing the number of up- (red) and downregulated (cyan) DEGs per treatment (rows) and incubation time (columns). Genes exhibiting differential expression in the DMSO control group at 48 or 72 h, relative to DMSO at 24 h, are highlighted in bold opacity. **(D)** Venn diagram illustrating the overlap of DEGs identified upon single Ibr, AS, or combined Ibr + AS treatment. The shades of red indicate the relative number of DEGs within each subset, with darker shades representing a higher number of DEGs. The combined Ibr + AS treatment accounted for the highest number of exclusively differentially expressed genes (1,460; 62%). Only 281 (12%) genes were differentially expressed in all three treatment groups.

Differential expression analysis comparing each treatment group at each incubation time to its respective control group (cells incubated only with DMSO for the same period) while adjusting for passage number identified a total of 2,336 DEGs (Supplementary Table 2). These genes were associated with adaptive immune response, lymphocyte-mediated immunity, lymphocyte proliferation, T-cell activation, negative regulation of megakaryocyte differentiation, glycolysis, leukocyte cell-cell adhesion, sterol metabolism, cytokine production, and apoptosis, among other biological processes (Supplementary Table 3). Hierarchical clustering and PCA of the expression values of the DEGs confirmed that the samples could be separated based on treatment type and incubation time (Figures 2A, B). A large portion of these genes (636; 27%) were also differentially expressed across incubation times for cells treated only with DMSO, albeit often discordantly (Figure 2C). Indeed, we identified 65 and 758 DEGs when comparing the control groups incubated for 48 and 72 h, respectively, to the one incubated for 24 h. These DEGs were associated with biological processes similar to the ones related to the treatments, including antigen processing and presentation, positive regulation of T-cell proliferation, regulation of leukocyte cell-cell adhesion, response to hypoxia, glycolysis, sterol metabolism, cytokine production, and apoptosis, confirming previous reports that DMSO has diverse effects on cellular activity [e.g., (60)] (Supplementary Table 3). These observations underscore the complexity of assessing drug-specific mechanisms in biological systems.

### 3.3 The combination of Ibrutinib and AS-605240 has synergistic effects on gene expression

While DEGs associated with single treatment with Ibr (792) or AS (383) only made up a small fraction of the DEGs (34% and 16%, respectively), the DEGs upon the combined Ibr + AS treatment (2,190) accounted for 94% of all DEGs (Figures 2C, D). Intriguingly, the total number of DEGs for both Ibr and AS single treatments increased over the incubation time, but we observed virtually no change in the total number of DEGs for the combined Ibr + AS treatment (Figure 2C). Additionally, single treatments induced a higher number of up-regulated than down-regulated DEGs at 24 h, with a subsequent shift toward down-regulation at 48 and 72 h, but the combined Ibr + AS treatment showed down-regulation as the dominant trend only at 72 h. As anticipated, most of the genes that were differentially expressed upon single treatment were also differentially expressed upon the combined Ibr + AS treatment: only 146 genes (6%) were exclusively differentially expressed upon either single Ibr (116) or AS (48) treatment (Figure 2D; Supplementary Figure 3). These genes were not associated with any specific biological processes. In contrast, the 281 (12%) genes differentially expressed upon all types of treatments were involved in antigen processing and presentation, sulfur amino acid transport, steroid and cholesterol steroid metabolic processes, and leukocyte-mediated cytotoxicity chemotaxis, steroid metabolic processes, and organic molecule transport, among other biological processes (Supplementary Table 3). Furthermore, the 1,460 genes that were only differentially expressed upon the combined Ibr + AS treatment were associated with biological processes including adaptive immune response, T-cell apoptotic process, leukotriene D4 metabolic process, leukocyte proliferation, glutathione catabolic process, and GTPase-mediated signal transduction (Supplementary Table 3). These findings are in agreement with synergistic effects observed for Ibr and AS in the proliferation and metabolic activity assays.

### 3.4 Co-expression network analysis identifies five gene clusters associated with single or combined treatments

**Figure 3:**
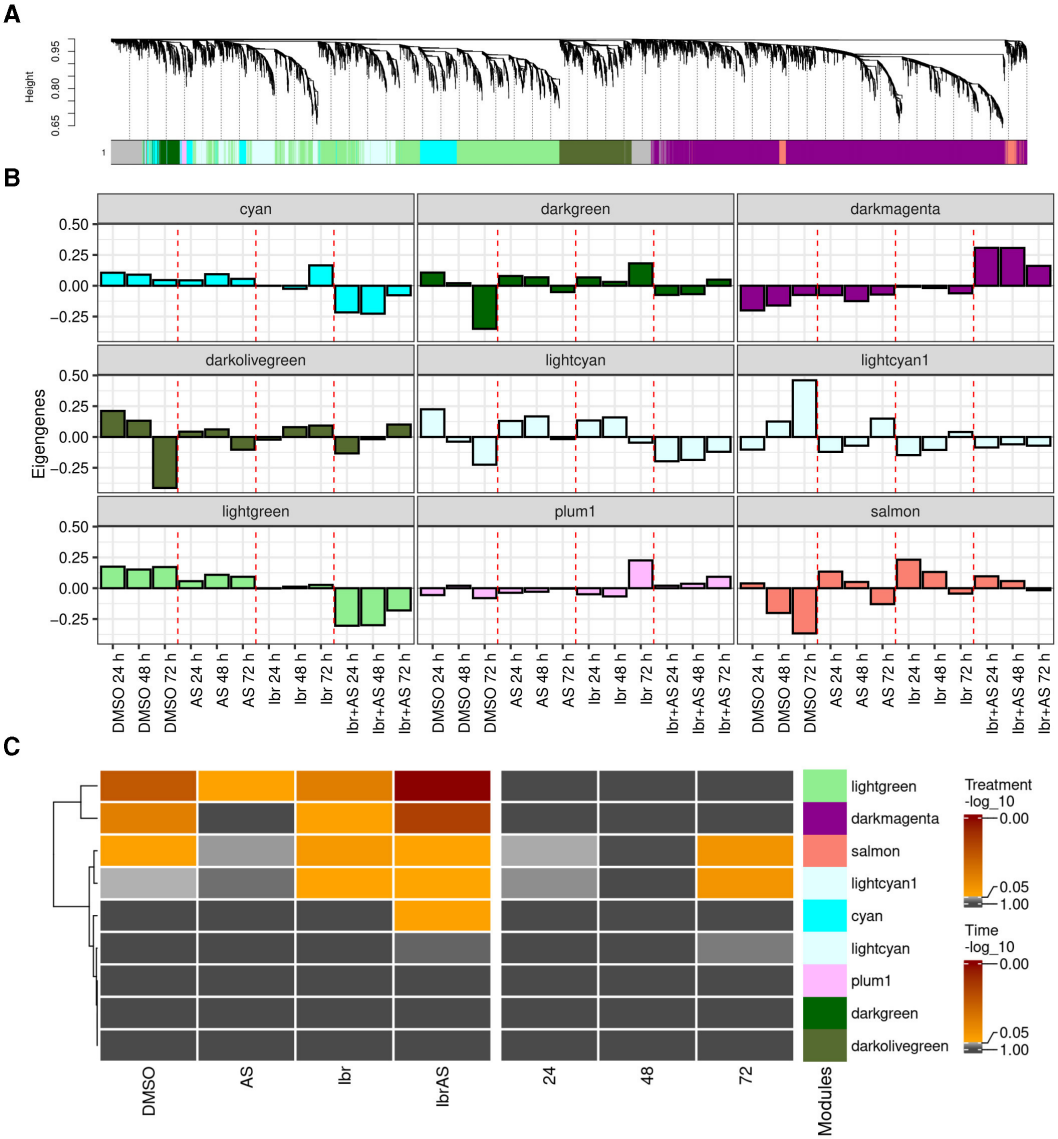
WGCNA revealed nine distinct gene modules, with five of them (*cyan, darkmagenta, lightcyan1, lightgreen, and salmon)* being potentially relevant to treatment response. **(A)** Average linkage hierarchical clustering dendrogram of the top two-third of the expressed genes with highest expression values. The color bars below the dendrogram (*x*-axis) show the module assignment of each gene, with gray representing unassigned genes. The *y*-axis (“Height”) represents the dissimilarity between genes, calculated based on their topological overlap (Supplementary Figures 1C, E). Higher heights indicate stronger dissimilarity. **(B)** Eigengenes of each treatment and incubation time averaged over passage reveal the expression profile of the combined Ibr + AS treatment to have the strongest (also negative) correlation to PC1 of the expression matrix of genes within the *darkmagenta, cyan*, and *lightgreen* modules compared to the eigengenes of the other treatments. **(C)** Heatmaps indicating linear regression-derived associations between the module eigengenes (*y*-axis) and treatment type and incubation times (*x*-axis). The color of the heatmaps represents the (–log_10_ value of the) false discovery rate (FDR).

To better characterize the transcriptional responses induced by the different treatments, we applied weighted gene co-expression network analysis (WGCNA) and identified nine clusters of co-expressed genes or *modules* (Section 2; Figure 3A). The number of genes in each module ranged from 75 to 3,888 genes, with a median of 650. A total of 626 genes were not co-expressed with other genes, and thus, not assigned to any module (*gray*).

In WGCNA, patterns inherent to modules are captured by their *eigengenes*, which can be thought of as weighted average expression profiles (Figure 3B) (32). Linear regression analysis of the eigengenes as a function of treatments and incubation times revealed that the *cyan* (*N* = 700), *darkmagenta* (*N* = 3,888)*, lightgreen* (*N* = 2,416), *lightcyan1* (*N* = 650), and *salmon* (*N* = 211) modules were potentially relevant to our research question, since they were associated with one or more treatments (Figure 3C). In particular, the *darkmagenta* module comprised *BTK*, the primary target of Ibrutinib. Compared to other treatments, genes in the *cyan, lightcyan1*, and *lightgreen* modules tended to exhibit lower expression levels upon combined Ibr + AS treatment. Conversely, genes within the *darkmagenta* and *salmon* modules demonstrated higher expression levels following the combined Ibr + AS treatment (Supplementary Figure 4). The *darkmagenta, lightgreen*, and *salmon* modules were associated with one or two of the single treatments and/or the combined Ibr + AS treatment. In contrast, the *cyan* module was exclusively associated with the combined Ibr + AS treatment and the *lightcyan1* module was only associated with the single Ibr treatment. The *lightcyan1* and *salmon* modules were the only modules associated with incubation time.

Functional enrichment analysis of potentially relevant modules suggested their involvement in distinct biological processes (Supplementary Table 3). Thus, the genes of the *cyan* module play roles in cellular metabolism and signaling and in protein synthesis and regulation). The genes in the *darkmagenta* module are related to immune function and T-cell function, cellular clearance and recycling processes, energy production, and mitochondrial function. The *lightcyan1* module contains genes important for cellular metabolism and homeostasis and protein trafficking and modification. The genes in the *lightgreen* module are associated with cell cycle regulation and DNA maintenance, and cellular organization and nucleotide metabolism. Finally, the *salmon* module was not associated with specific biological processes, indicating its implication in diverse cellular functions.

In summary, this analysis confirms that Ibr and AS may have complementary mechanisms of action.

### 3.5 A substantial portion of DEGs were found within modules potentially relevant to treatment responses

**Figure 4:**
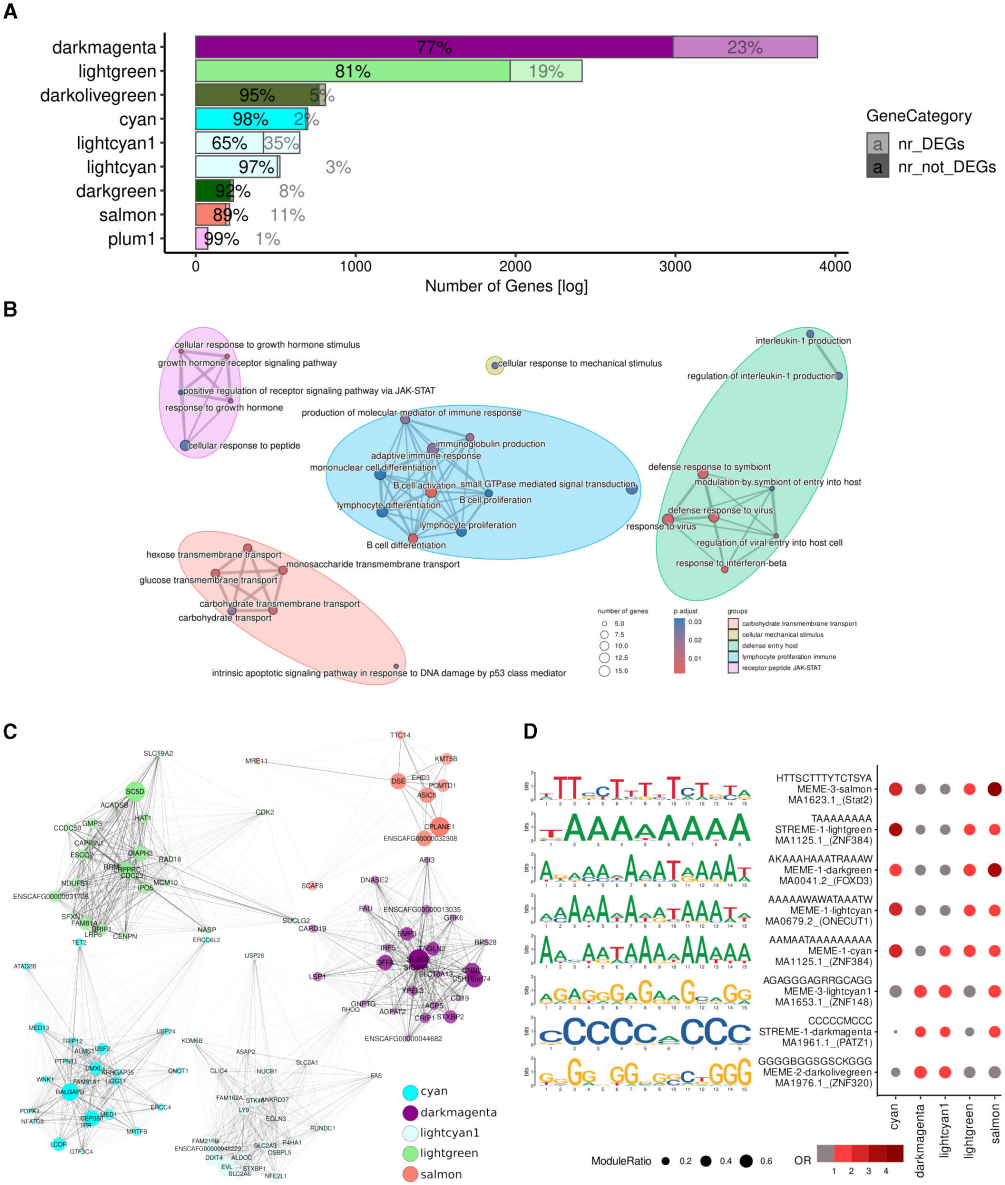
A large proportion of the DEGs were found within potentially relevant modules. **(A)** Overlaps between module member genes (solid) and DEGs (low opacity). **(B)** GO terms of 1,617 genes which were either differentially expressed or in one of the potentially relevant modules identified by WGCNA (Figure 3C; cyan, *darkmagenta, lightcyan1, lightgreen*, and *salmon*). **(C)** Dot plot of the motifs overrepresented among the promoters of DEGs in potentially relevant modules. The size of the circle corresponds to the fraction of DEG promoters in the module with instances of the motif; odds ratios relative to all DEGs in potentially relevant modules are indicated in red. **(D)** Top 25 hubs of each module, selected based on module eigengene-based connectivity (kME), are represented as a graph with forced-directed layout as proposed by Kamada and Kawai (50) using the R plugin RCy3 (51) for constructing networks with Cytoscape (49). Each node represents a hub and edge thickness indicates the TOM similarity. Node size indicates its module eigengene-based connectivity (kME), i.e., the correlation between the expression profile of a gene and the module eigengene and node opacity corresponds to its intramodular connectivity (kWithin).

Of the 9,512 genes in the (non-gray) modules, 1,693 (18%) were differentially expressed. Among them, virtually all (1,617, 95%) were members of modules potentially relevant to treatment response (*cyan, darkmagenta, lightcyan1, lightgreen*, and *salmon*) (Figure 4A; Supplementary Figure 5). Conversely, four of the five modules exhibiting the greatest proportion of DEGs were potentially relevant to treatment response: *lightcyan1* (227 DEGs, 35% of its members), *darkmagenta* (904 DEGs, 23% of its members), *lightgreen* (450 DEGs, 19% of its members), and *salmon* (211 DEGs, 11% of its members; Figure 4A; Supplementary Figure 5). DEGs in relevant modules were related to biological processes such as immune response, leukocyte proliferation and apoptosis, acetyl-CoA metabolic process, and sterol biosynthesis (Figure 4B).

To better understand the molecular mechanisms underlying the different treatment responses, we then examined the *hub* genes of each of the potentially relevant modules (Figure 4C), in particular those which were also differentially expressed (Supplementary Table 4). Hubs are genes involving a large number of interactions in a network or part thereof, and are traditionally considered important because changes in their expression may result in changes in the expression of all their interaction partners (61, 62). The *cyan, darkmagenta, lightcyan1, lightgreen*, and *salmon* modules contained 2, 143, 31, 47, and 3 differentially expressed hubs, respectively. Notably, the *top* hub (i.e., those with the highest number of interactions; Section 2) of the *darkmagenta, lightcyan1*, and *lightgreen* modules were differentially expressed in response to one or more of treatments (Supplementary Table 4). The top hub of the *darkmagenta* module was *ACP5* (Acid Phosphatase 5, Tartrate Resistant), a gene encoding a glycoprotein that catalyzes the conversion of orthophosphoric monoester to alcohol and orthophosphate, and can promote cell proliferation and tumorigenesis via the *FAK/PI3K/AKT* signaling pathway (63, 64). The top hub of the *lightcyan1* module was *EVL* (Enah/Vasp-like), which is predicted to enable SH3 domain binding activity and profilin binding activity (65). EVL suppresses cell migration and low *EVL* expression has been correlated with advanced stage of breast cancer and poor patient outcome (66). Lastly, the top hub gene of the *lightgreen* module was *SC5D* (Sterol-C5-Desaturase), which encodes one of the key enzymes of cholesterol biosynthesis (67). Although inconclusive, loss of function in SC5D has been suggested as a contributor to cancer progression (68, 69).

Motif analysis conducted on the promoters of the DEGs within potentially relevant modules revealed enrichment for C/G- and A/T-rich binding sites (Figure 4D). Specifically, DEG promoters in the *cyan* and *lightgreen* modules were enriched for motifs consistent with the binding site of the transcription factor ZNF384 (Zinc Finger Protein 384); DEG promoters in the *darkmagenta* module were characterized by the presence of binding sites putatively recognized by PATZ1 (POZ/BTB and AT Hook Containing Zinc Finger 1); DEG promoters in the *lightcyan1* module featured binding sites for ZNF148 (Zinc Finger Protein 148); and finally, those in the *salmon* module exhibited binding sites for STAT2 (Signal Transducer And Activator Of Transcription 2). These transcription factors have been implicated in various aspects of cancer biology, although specific studies in lymphoma are limited. ZNF384 encodes a zinc finger protein and is a recurrent fusion partner in various leukemias [e.g., (70–72)]. Furthermore, ZNF384 appears to regulate the expression of extracellular matrix genes like *MMP1, MMP3, MMP7*, and *COL1A1* in multiple cancer types (73–75). PATZ1 inhibits the DNA binding of TP53, thereby controlling cell proliferation and DNA damage responses (76), and has been suggested as a tumor suppressor and prognostic marker of DLBCLs (77). Increased expression of ZNF148 is associated with signaling pathways important for proliferation, embryogenesis, differentiation, growth arrest, and apoptosis (78, 79), and has been suggested to cooperate with other transcription factors in hematopoietic cells (80). Lastly, STAT2 is a member of the STAT family of transcription factors that mediate innate antiviral activity (81) and are involved in the regulation of cell survival and proliferation; dysregulation of STAT transcription factors—in particular, of STAT3—has been associated with the pathogenesis of various lymphomas (82).

Lastly, to uncover the mechanisms underlying the unique effects of the combined Ibr + AS treatment, we centered our attention on the 12 DEGs in the *cyan* module (2% of its members)—the only module exclusively associated with the combined Ibr + AS treatment: *NHSL1, PALM2AKAP2, INTU, NUTM1, PAG1, ZNF106, ZMAT3, PRKAR2A, CLCN5, STON2, ACACA*, and *LCOR*. Suggesting their potential roles as central regulators, a majority of these genes (*PALM2AKAP2, PAG1, ZNF106, PRKAR2A, STON2, ACACA, and LCOR*; Table 1) were strongly connected with other genes within the same module (above the 85th percentile). Except for *NUTM1*, all of them tended to be downregulated following every treatment. Interestingly, the downregulation of these genes was substantially more pronounced upon the combined Ibr + AS treatment. Three of these genes: *PAG1* (Phosphoprotein associated with glycosphingolipid-enriched microdomains 1), *PRKAR2A* (Protein Kinase cAMP-Dependent Type II Regulatory Subunit Alpha), and *ACACA* (Acetyl-CoA Carboxylase Alpha) are particularly noteworthy due to their involvement in the PI3K pathway (83–87).

**Table 1:** Putative key effectors of the combined Ibr + AS response.

**Selection**	**Gene**	**log**_**2**_ **FC**			**Module**	**k_%_**
		***AS***	***Ibr***	***AS** + **Ibr***		
Hub genes of potentially relevant modules	*ACP5*	0.09	0.73^*^	2.07^*^	*darkmagenta*	100th
	*EVL*	−0.66^*^	−0.74^*^	−0.69^*^	*lightcyan1*	100th
	*SC5D*	−0.32	−0.71^*^	−1.85^*^	*lightgreen*	100th
	*RALGAPB*	−0.08	−0.25	−0.68^*^	*cyan*	100th
	*DSE*	0.15	0.41^*^	0.36^*^	*salmon*	99th
DEGs in the cyan module	*NHSL1*	−0.22	−0.45	−1.11^*^	*cyan*	48th
	*PALM2AKAP2*	−0.38	−0.81^*^	−1.81^*^	*cyan*	93rd
	*INTU*	−0.06	−0.11	−1.04^*^	*cyan*	64th
	*NUTM1*	0.02	−1.28^*^	−0.92	*cyan*	4th
	***PAG1***	−0.39	−0.81^*^	−1.09^*^	*cyan*	86th
	*ZNF106*	−0.31	−0.48^*^	−1.16^*^	*cyan*	97th
	*ZMAT3*	−0.51	−0.73	−1.48^*^	*cyan*	58th
	***PRKAR2A***	−0.12	−0.49^*^	−1.24^*^	*cyan*	88th
	*CLCN5*	−0.53	−0.51	−1.12^*^	*cyan*	52nd
	*STON2*	−0.26	−0.93^*^	−2.08^*^	*cyan*	95th
	***ACACA***	−0.52	−0.89^*^	−1.44^*^	*cyan*	93rd
	*LCOR*	−0.08	−0.28	−1.02^*^	*cyan*	99th
Genes in the BCR pathway	*BTK*	0.18^*^	0.44^*^	0.65^*^	*darkmagenta*	87th
	***FOS***	−0.32	−1.44^*^	−1.60^*^	*lightgreen*	24th
	*PLCG2*	−0.04	0.14	0.11	*darkgreen*	42nd
	*LYN*	0.03	0.36^*^	0.42^*^	*darkmagenta*	80th
	*SYK*	−0.08	0.21^*^	0.69^*^	*darkmagenta*	93rd
	*PIK3CA*	0.22	0.35	−0.12	*cyan*	34th
	*PIK3CB*	0.04	−0.04	−0.31^*^	*lightcyan*	54th
	*PIK3CD*	−0.30^*^	−0.26^*^	0.13	*darkmagenta*	39th
	*PIK3CG*	−0.46	−0.85^*^	−0.38	*lightcyan1*	10th
	*PIK3R1*	0.06	0.05	0.14	*darkgreen*	53rd
Genes in the the BCR pathway	*PIK3R2*	−0.20	−0.26	0.27	*NA*	NA
	*PIK3R3*	−0.17	−0.37	−1.53^*^	*lightgreen*	96th
	*PIK3R5*	0.27	0.62^*^	1.35^*^	*darkmagenta*	100th
	*PIK3R6*	0.57	1.53^*^	2.18^*^	*darkmagenta*	84th
	*AKT1*	−0.04	−0.13	0.26^*^	*darkmagenta*	60th
	*AKT2*	−0.01	−0.01	−0.01	*darkmagenta*	7th
	*AKT3*	−0.15	−0.36	1.33^*^	*darkmagenta*	70th
	*MYC*	−0.10	−0.25	0.99^*^	*darkmagenta*	86th
	*BCL2*	−0.05	−0.09	−0.78^*^	*lightgreen*	48th
	*PRKCB*	0.03	0.31^*^	0.57^*^	*darkmagenta*	88th
	*CHUK*	0.14	0.07	−0.30^*^	*lightgreen*	85th
	*IKBKB*	0.03	0.29^*^	0.79^*^	*darkmagenta*	95th
	*IKBKG*	−0.10	−0.16	−0.04	*darkgreen*	49th
Genes in the PI3K pathway	*PIK3CG*	−0.46	−0.85^*^	−0.38	*lightcyan1*	16th
	*PIK3R5*	0.27^*^	0.62^*^	1.35^*^	*darkmagenta*	100th
	*PIK3R6*	0.57^*^	1.53^*^	2.18^*^	*darkmagenta*	85th
	*AKT1*	−0.04	−0.13	0.26^*^	*darkmagenta*	60th
	*AKT2*	−0.01	−0.01	−0.01	*darkmagenta*	7th
	*AKT3*	−0.15	−0.36	1.33^*^	*darkmagenta*	70th
	***PRKCA***	−0.14	−0.08	−0.67^*^	*cyan*	92nd

Curated list of genes, including: top hub members identified through network analysis, DEGs in the cyan module, genes involved in the BCR signaling pathway, genes that are central nodes of the PI3K-AKT pathway. DEGs are highlighted in pink.

Genes identified as the most likely central effectors of the synergistic effect of Ibr + AS are noted in magenta bold font.

^*^P-value < 0.05 for AS, Ibr, and AS+Ibr compared to DMSO at 48 h.

log_2_FC: log_2_ FCs of AS, Ibr, and AS + Ibr compared to DMSO at 48 h; module: module to which; Gene is a member of; kme: module eigengene-based connectivity; kWithin: intramodular connectivity; k_%_: percentile of kWithin.

### 3.6 Ibr + AS targets *FOS* in the BCR signaling and AKT3 in the PI3K/AKT signaling pathways

**Figure 5:**
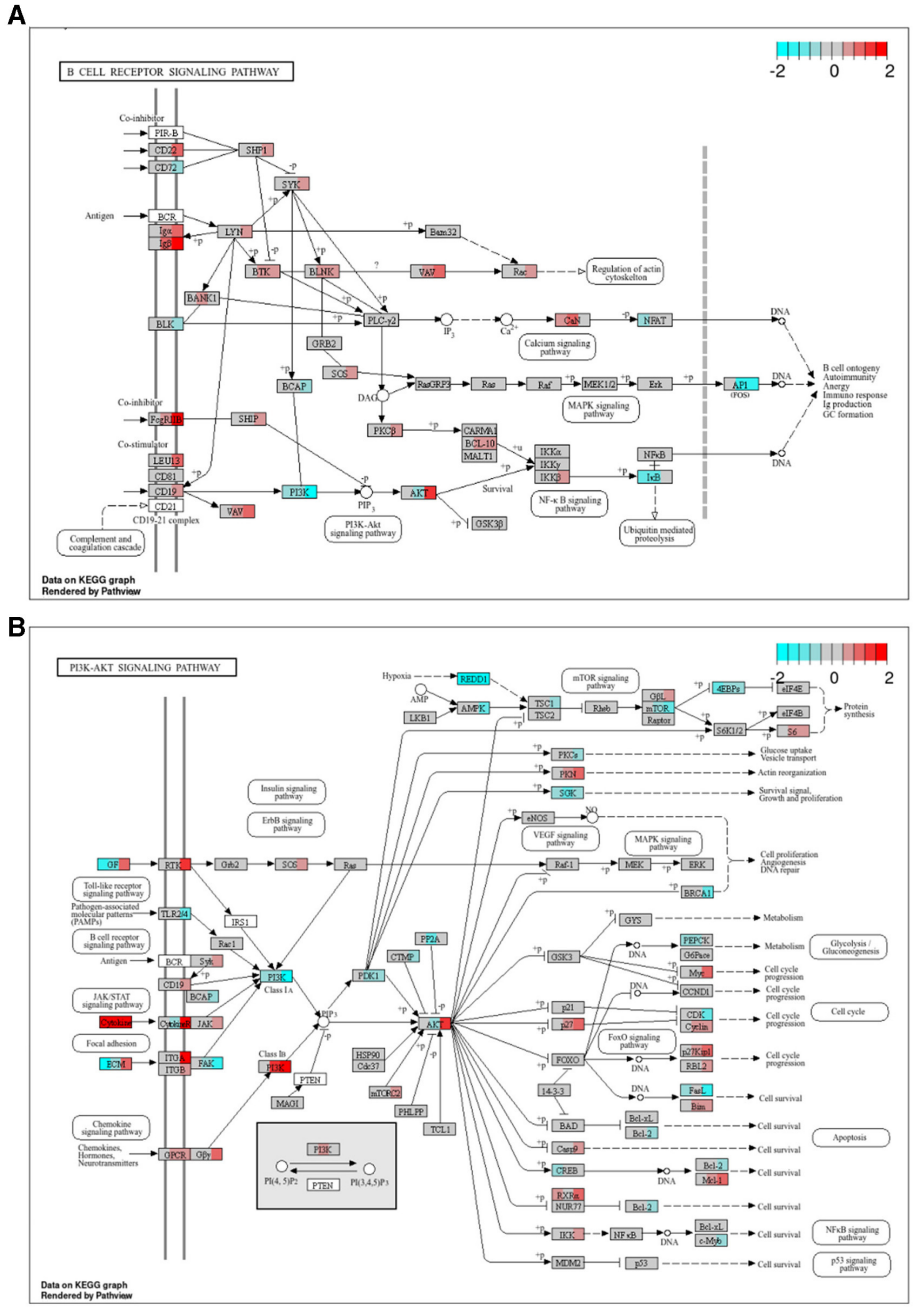
Combined Ibr + AS treatment modulates both upstream and downstream effectors of the BCR (A, B) PI3K-AKT signaling pathways. KEGG (58, 59) pathway graphs rendered by “pathview” (57) R package for the “B-cell receptor signaling pathway” (hsa04662) and “PI3K-Akt signaling pathway” (hsa04151). Rectangular boxes represent proteins and circles, metabolites. Boxes with rounded corners contain references to other pathways. Rectangular boxes are divided into three segments; from left to right, these segments represent: single treatment with AS, single with Ibr, and combined treatment with Ibr and AS. Segments are colored based on the log_2_ FCs relative to the DMSO control of the RNA transcripts of the respective proteins. Proteins encoded by genes that are not differentially expressed nor members of a potentially relevant module (*lightgreen, salmon, darkmagenta, lightcyan1* or *cyan*) are shown in gray. White rectangular boxes correspond to RNA transcripts that were considered non-expressed or undetected. Canine genes (Entrez IDs) were mapped to their human orthologs. **(A)** Despite reduced protein expression of BTK, Ibrutinib treatment led to increased BTK mRNA levels, suggesting compensatory mechanisms. Nevertheless, *FOS*, the gene encoding a subunit of the AP-1 transcription factor, a downstream effector of the BCR pathway, was significantly downregulated. Note that the gray box labeled “PI3K” represents the catalytic (PIK3CA, PIK3CB, PIK3CD, and PIK3CG) and regulatory subunits (PIK3R1, PIK3R2, PIK3R3, PIK3R5, and PIK3R6) of the PI3K enzyme complex. **(B)** AS treatment selectively inhibited PI3K activation. We see strongest downregulation of PI3K class IA and upregulation of PI3K class I B in the combined treatment group. PI3K class I B constitutes the main target of AS, PIK3CG, as well as its regulatory subunits PIK3R5 and PIK3R6 which Ibr + AS seem to especially be influencing. Downstream thereof is AKT, an upstream regulator of MYC and BCL2. Although the isoform AKT3 was upregulated in Ibr + AS, MYC expression was substantially higher and BCL2 expression was substantially lower in the combined treatment.

To obtain insights into the biological context of the observed gene expression changes, we mapped the log_2_ FCs relative to the DMSO control at 48 h of genes that were differentially expressed genes upon AS, Ibr, or combined Ibr + AS treatment onto the BCR and PI3K-AKT signaling KEGG pathways (Figure 5, Table 1; Section 2). Specifically, we focused on the samples incubated for 48 h to maximize the likelihood that the observed biological effects were primarily due to the treatment and not off-target factors (Figures 2A, C).

Among the genes within the BCR signaling pathway, only four–*AKT3, FOS* (Fos Proto-Oncogene, AP-1 Transcription Factor Subunit), *CD79A* and *CD79B*—were differentially expressed relative to the DMSO control at 48 h. Additionally, 66 genes were members of potentially relevant modules, many of which showed enhanced differential expression upon combined Ibr + AS treatment compared to single-agent treatments.

While we previously confirmed that BTK protein expression was reduced in CLBL-1 upon single Ibr and combined Ibr + AS treatments (83), RNA-seq analysis revealed a trend toward *BTK* mRNA upregulation. Moreover, several genes involved in the BCR signaling pathway, including *CD79A, CD79B, LYN*, and *SYK*, exhibited expression patterns consistent with elevated BTK activity. Despite these observations, *FOS*, the gene encoding one of the two subunits of the Protein-1, AP-1, a downstream effector of the BCR pathway, was significantly downregulated upon both single Ibr and combined Ibr + AS treatment. Notably, the downregulation of *FOS* was slightly more pronounced upon the combined Ibr + AS treatment.

Similar to *BTK*, also AKT exhibited discordance between protein and transcript levels—in particular, for *AKT3*. While AKT is another key downstream effector of the BCR signaling pathway, it can be activated independently, primarily by the PI3K-AKT pathway.

Within the PI3K-AKT signaling pathway, 23 genes were differentially expressed relative to the DMSO control at 48 h, and 130 were members of potentially relevant modules. Although most genes within this pathway exhibited similar expression changes following treatment with Ibr alone or Ibr + AS, key exceptions revealed a complex interplay between cell cycle regulation, survival, and metabolic processes (Table 1). Specifically, combined Ibr+AS treatment resulted in greater upregulation of *MYC* and *CDKN1B* compared to Ibr alone, suggesting a potential acceleration of cell cycle progression that could contribute to tumor growth and therapeutic resistance. Conversely, combined Ibr + AS treatment resulted in greater downregulation of genes involved in cell survival such as *FASLG, BCL2*, and *MYB*, likely enhancing the efficacy of cell death-inducing treatments. Furthermore, the more pronounced downregulation of *BRCA1*, a key regulator of DNA repair and cell proliferation, suggests increased vulnerability to DNA-damaging agents upon combined Ibr + AS treatment. Consistent with these findings, the gene encoding protein kinase C alpha, *PRKCA*, which is involved in glucose uptake and metabolism, also exhibited enhanced downregulation upon combined Ibr + AS treatment, perhaps indicating a metabolic shift that may impair tumor growth and survival by reducing energy availability. Finally, consistent with *AKT3* expression upregulation, we observed downregulation of *NFKBIE*, encoding the Inhibitor of kappa B epsilon (IκBε), which prevents the nuclear translocation of NF-κB (88).

These findings underpin the complexity of BCR signaling and the challenges of predicting downstream effects based solely on single gene expression changes, but also highlight differences between the transcriptome and the proteome.

## 4 Discussion

Ibrutinib, a potent BTK inhibitor, shows promise in treating canine B-cell lymphoma. However, limitations like off-target effects, low tolerability, resistance development, and high relapse rates underscore the necessity for novel therapeutic strategies for improved patient outcomes. Second and third-generation BTK inhibitors have been developed to address some of these shortcomings. For instance, acalabrutinib, a second-generation BTK inhibitor, exhibits a favorable toxicity profile and at least equivalent efficacy to Ibrutinib in chronic lymphocytic leukemia (CLL) patients, and is FDA-approved for its treatment (89). Furthermore, to enhance therapeutic efficacy and provide a more robust treatment approach, Ibrutinib has been combined with other agents, such as the immunomodulator lenalidomide or the monoclonal antibody against the B-cell surface protein CD20 rituximab (90). Given the complementary mechanisms of action targeting key survival pathways in B-cells, combining Ibrutinib with a PI3K inhibitor holds significant promise for improved therapeutic outcomes. Nonetheless, reports on the combined application of BTK and PI3K inhibitors in canine B-cell lymphoma remain limited. Building upon our previous research, this study aimed to investigate the combined effects of these two kinase inhibitors at the transcriptomic level. To this end, we performed RNA-seq on CLBL-1, a canine B-cell line derived from a DLCL, treated with AS-605240 (AS), Ibrutinib (Ibr), or the combination of the two (Ibr + AS) for 24, 48, and 72 h.

Employing a combined bioinformatics approach involving differential expression analysis (DEA) and weighted gene co-expression network analysis (WGCNA), this study confirmed a substantially greater number of differentially expressed genes resulting from the combined treatment of Ibrutinib and AS-605240, and identified *PAG1, PRKAR2A, ACACA, FOS*, and *PRKCA* as key candidate genes contributing to the observed synergistic effect. DEA uses statistical modeling to determine whether a gene is differentially expressed while accounting for specific features of each sample and for the sparsity of data associated with RNA-Seq experiments. Genes are studied individually in the context of DEA, although they are known to operate in networks showing correlated expression patterns. Therefore, approaches like WGCNA investigate gene expression data sets in the context of network theory. The resulting network is derived from pairwise correlations between gene expression levels from which genes can be clustered into modules by hierarchical clustering. We exploited the complementarity of DEA and WGCNA and assumed genes identified by both methods are highly likely to be of functional significance (91).

In line with the experiments testing proliferation and metabolic activity of CLBL-1, we found that treatment type and incubation time significantly influenced gene expression patterns. DEA revealed 2,336 DEGs across all treatment groups when compared to DMSO at the respective incubation times, of which 94% were attributed to the combined treatment Ibr + AS. Interestingly, a large proportion of these genes were differentially expressed when comparing different incubation times for the control group, which was only treated with DMSO. Because DMSO easily penetrates biologic membranes, it is a commonly used solvent and delivery system for therapeutic agents that are not water soluble (92). Further, DMSO is commonly used for long time storage of viable cells, preventing freeze-thaw-induced cell disruption and allowing the recultivation of primary cells and cell lines after prolonged storage. The observed increase in DEGs with incubation time may be attributable to prolonged culture without media replenishment, potentially resulting in increased cellular necrosis. Nevertheless, these findings highlight the importance of considering DMSO's potential effects on gene expression, particularly in long-term experiments, when interpreting results from studies using this solvent. Co-expression analysis identified nine gene modules, five of which were potentially associated with one or more treatments and comprised 95% of the DEGs. Promoters of DEGs within potentially relevant modules were enriched for G/C- and A/T-rich motifs. We found binding sites enriched for zinc fingers PATZ2, which inhibits DNA binding of TP53 and is therefore a potential tumor suppressor, ZN384, a putative regulator of extracellular matrix genes in various cancers (93–95), and the STAT2 transcription factor, which is involved in the regulation of cell survival and proliferation. Notably, the *cyan* module was the only one exclusively associated with the combined Ibr + AS treatment, encompassing 12 differentially expressed genes (DEGs) among its 700 members. Among these DEGs, three are noteworthy because they are linked to key cellular processes known to be relevant in lymphoma, including modulation of BCR and PI3K/AKT/mTOR signaling: *PAG1* (Phosphoprotein associated with glycosphingolipid-enriched microdomains 1) encodes a transmembrane adaptor protein that directs the intracellular localization of kinases of the Src tyrosine kinase family—like LYN and SYK—to negatively regulate their activity (96), modulating the signaling cascade initiated by BCR engagement and acting as a negative regulator of the PI3K/AKT/mTOR pathway (97, 98). *PRKAR2A* (Protein Kinase cAMP-Dependent Type II Regulatory Subunit Alpha) has been implicated in the regulation of the PI3K-AKT pathway through its role in cAMP signaling (99). *ACACA* (Acetyl-CoA Carboxylase Alpha) is primarily involved in fatty acid metabolism, its regulation has been shown to indirectly affect the PI3K-AKT pathway in the context of metabolic reprogramming in cancer cells (100).

Inspection of expression changes of members of the BCR pathway revealed relatively few DEGs within this pathway, and a striking discordance between *BTK* mRNA levels, which trended upwards, in contrast to our previously reported reductions in BTK protein (96). This discrepancy underscores the importance of integrating proteomic data with transcriptomic analyses to gain a comprehensive understanding of cellular processes, since mRNA abundance does not always reflect protein levels. Moreover, the expression patterns of *CD79A, CD79B, LYN*, and *SYK* suggest increased BTK activity despite the observed protein reduction, which could be explained by mechanisms such as post-translational modifications (e.g., phosphorylation), alterations in related signaling pathways, and feedback loops. Nevertheless, the observed downregulation of *FOS*, a key component of the AP-1 transcription factor, suggests a potential disruption of downstream signaling from the BCR. Remarkably, the downregulation of FOS was slightly more pronounced upon the combined Ibr + AS treatment relative to treatment with Ibr alone, suggesting a possible mechanism underlying the synergistic effects observed for this therapeutic approach. This aligns with recent studies demonstrating that inhibiting FOS, such as in combination with a histone deacetylase inhibitor, can effectively kill DLBCL cells (101). Within the PI3K-AKT pathway, the combined Ibr + AS treatment elicited a complex transcriptional response, impacting crucial processes such as cell cycle regulation, survival, and metabolism. Similar to BTK, AKT also exhibited a discordance between protein and transcript levels, but a key finding potentially explaining the synergistic effects of the combined Ibr + AS treatment was the enhanced downregulation of *PRKCA* compared to the treatment with Ibr alone. *PRKCA* encodes a member of the PKC family of enzymes and plays a significant role in regulating glucose uptake and metabolism, thereby increasing or inhibiting the activity of the PI3K-AKT pathway depending on the context (102).

While conducted in cell lines and thus subject to the limitations inherent in such models, this study offers a framework for understanding the synergistic effects of Ibrutinib and AS-605240, and highlights potential therapeutic strategies for canine B-cell lymphoma. The significant impact of B-cell lymphoma on canine welfare and the established link between pet ownership and human wellbeing (103–105) underscore the importance of developing effective therapies for this disease. Furthermore, the recognized relevance of canine B-cell lymphomas as a valuable model for human DLBCL highlights the translational potential of our findings. This warrants further investigation into the clinical efficacy of treatments combining tyrosine kinase inhibitors in both canine and human patients.

## Data Availability

The datasets presented in this study can be found in online repositories. The names of the repository/repositories and accession number(s) can be found below: https://www.ncbi.nlm.nih.gov/geo/query/acc.cgi?acc=GSE287971.
