# Supplementary material for: Transcriptomic profiling in canine B-cell lymphoma supports a synergistic effect of BTK and PI3K inhibitors
Source: Front Vet Sci. 2025 Apr 25;12:1577028. doi: 10.3389/fvets.2025.1577028 (PMC12063356; doi:10.3389/fvets.2025.1577028)
Supplement: Supplementary file 5 [file Data_Sheet_1.pdf]

# Transcriptomic profiling in canine B-cell lymphoma supports a synergistic effect of BTK and PI3K inhibitors

**Xenia Lainscsek[1], Weibo Kong[2], Barbara Rütgen[3], Julia Beck[4], Bertram Brenig[5], Ingo Nolte[6], Hugo Murua Escobar[2,7,*], Leila Taher[1,8,*]**

[1]Institute of Biomedical Informatics, Graz University of Technology, Graz, Austria

[2]Clinic for Hematology, Oncology and Palliative Care, Rostock University Medical Center, University of Rostock, Rostock, Germany

[3]Department for Pathobiology, Clinical Pathology, University of Veterinary Medicine Vienna, Vienna, Austria

[4]Chronix Biomedical GmbH, 37073, Göttingen, Germany

[5]University of Göttingen, Institute of Veterinary Medicine, Göttingen, Germany

[6]Small Animal Clinic, University of Veterinary Medicine Hannover, Hannover, Germany

[7]Institute of Medical Genetics, Rostock University Medical Center, University of Rostock, Rostock, Germany

[8]Institute for Biostatistics and Informatics in Medicine and Ageing Research, Rostock University Medical Center, University of Rostock, Rostock, Germany

*** Correspondence:**
Hugo Murua Escobar
hugo.murua.escobar@med.uni-rostock.de
Leila Taher
leila.taher@tugraz.at

* These authors contributed equally to this work and share last authorship.

## *Supplementary Material*

# 1 Supplementary Tables Legends

**Supplementary Table 1. Meta data of RNA-sequencing samples.**

**Supplementary Table 2. Results of the differential gene expression analysis across all comparisons of interest.** Each sheet corresponds to a specific comparison (detailed in the sheet name). Columns represent: Ensembl gene ID, gene symbol, $\log_2$ fold-change (magnitude and direction of expression change), (Benjamini-Hochberg) adjusted *P*-value, and weighted gene co-expression network analysis (WGCNA) membership. 1) AS compared to DMSO at respective incubation times. 2) Ibr compared to DMSO at respective incubation times. 3) Ibr+AS compared to DMSO at respective incubation times. 4) DMS0 48 and 72 h compared to DMSO at 24h

**Supplementary Table 3. Functional enrichment analysis of differentially expressed genes and genes in potentially relevant weighted gene co-expression network analysis (WGCNA) modules.** Columns include: ID (GO term ID); Description (brief description of the term); GeneRatio (number of dataset genes associated with the term divided by the total number of dataset genes); BgRatio (number of background genes associated with the term divided by the total number of background genes); pvalue (probability of observing the observed enrichment by chance); p.adjust (p-value adjusted for multiple testing using the Benjamini-Hochberg method); qvalue (minimum false discovery rate at which the enrichment is considered significant); geneID (list of dataset genes associated with the term); and Count (number of dataset genes associated with the term). 1) Gene Ontology (GO) biological process (BP) terms enriched among the DEGs identified in each treatment group at each incubation time relative to cells incubated only with DMSO for the same period. 2) GO BP terms among the DEGs identified in cells incubated only with DMSO at 48h and 72h relative to cells incubated only with DMSO at 24h. 3) GO BP terms enriched among the DEGs common to all three treatment groups for any incubation time relative to cells incubated only with DMSO. 4) GO BP terms of the genes which were exclusively differentially expressed upon the combined treatment Ibr+AS. 5) GO BP terms of genes contained in the cyan module. 6) GO BP terms of genes contained in the darkmagenta module. 7) GO BP terms of genes contained in the lightcyan1 module. 8) GO BP terms of genes contained in the lightgreen module. 9) GO BP terms of genes contained in the salmon module.

**Supplementary Table 4. List of genes belonging to each module identified by the weighted gene co-expression network analysis (WGCNA).** Each gene's *kme*, *kwithin*, $\log_2$ fold-change, and adjusted *P*-values are also given.

## 2 Supplementary Figures

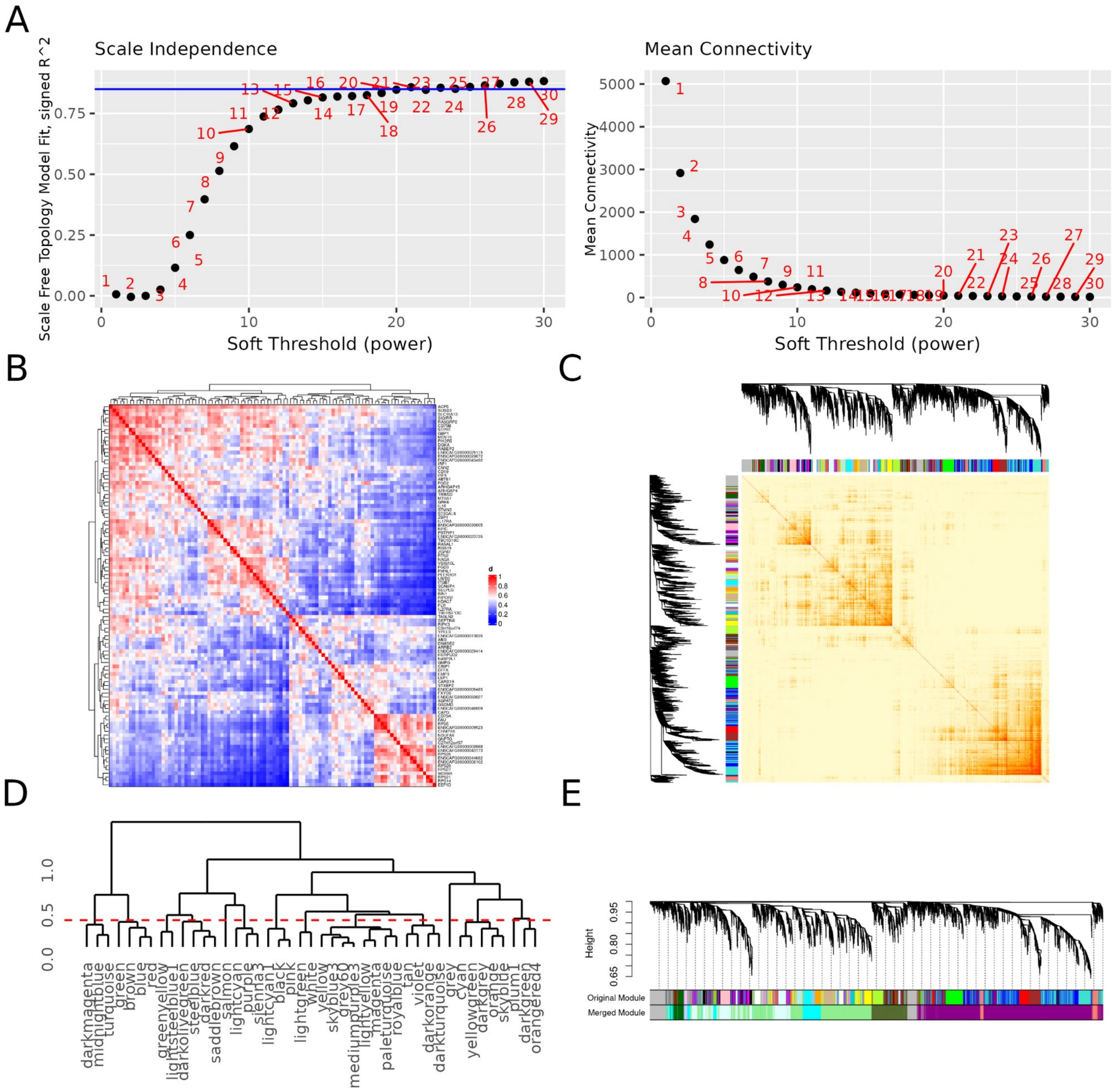


**Supplementary Figure 1. Weighted gene co-expression network analysis**. (**A**) Network topologies "scale independence" and "mean connectivity" for various soft-thresholding powers reveals β=21 as the value which maximizes $R^2$ while maintaining a high mean number of connections. (**B**) Connection strengths between the 100 most variable genes (standard deviation across samples) are reported by their adjacency matrix. (**C**) Topological overlap matrix and resulting hierarchical clustering dendrogram reveals 9 distinct gene modules. (**D**)

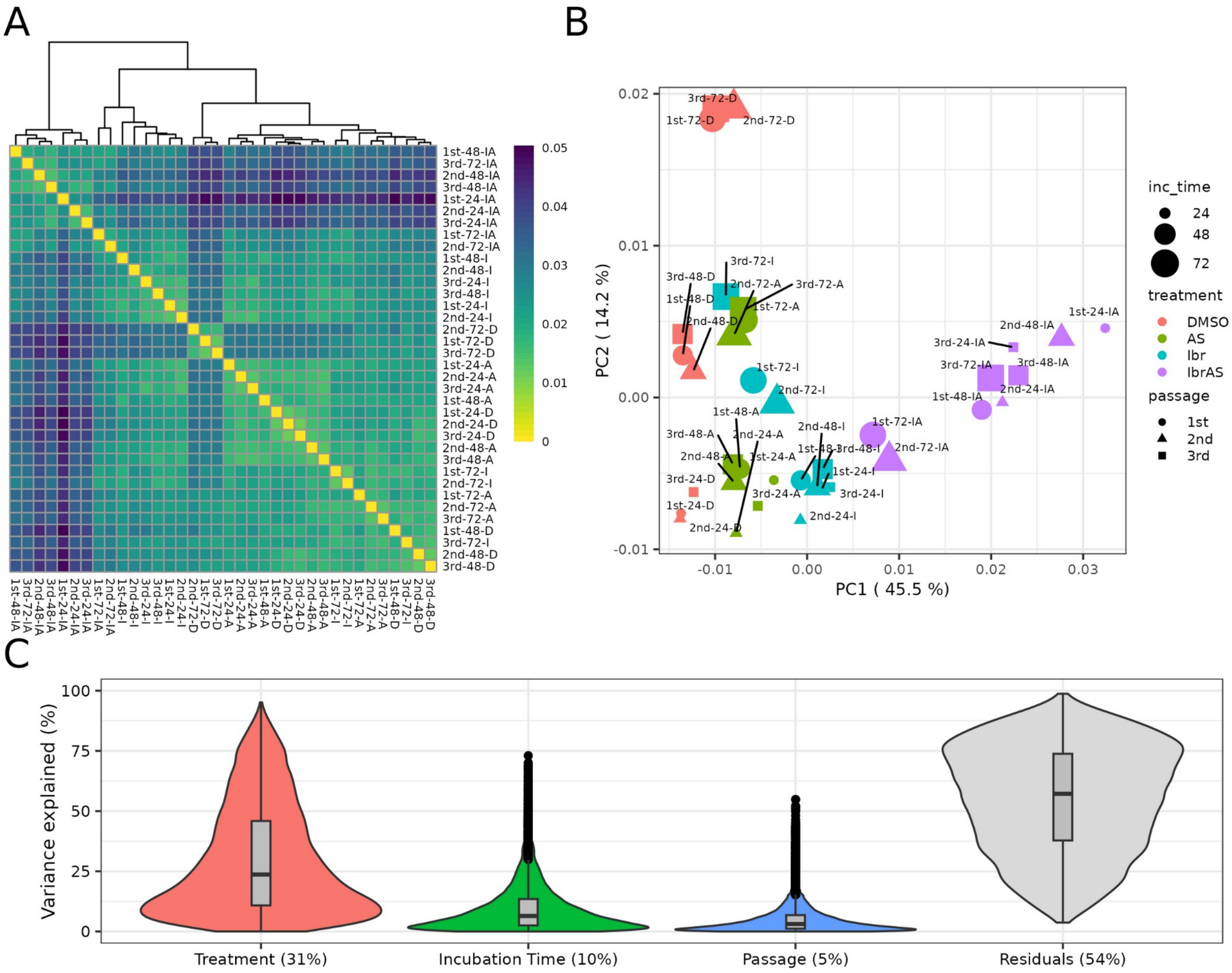


**Supplementary Figure 2. Treatment type is the main driver of variation in gene expression between samples, followed by incubation time.** (**A**) The heatmap of the dissimilarity matrix obtained from 15,237 expressed genes clusters the 36 samples according to the complete linkage method (covering 3 inhibitor-treatment types and one control, 3 inhibitor-incubation times, and 3 cell passage numbers). The color scale represents the Euclidean distance between gene expression profiles of respective samples. The third passage of the sample treated for 72h with Ibrutinib manifests as an outlier. (**B**) First two principal components of expression values of each sample explain 45.5% and 14.2% of variance. (**C**) The violin plot generated by the "variancePartition" R package summarizes the contribution of treatment type, incubation time, and cell passage number to the variation in the gene expression values. Treatment type (31%) constitutes for the most variation followed by incubation time (10%) and passage (5%).

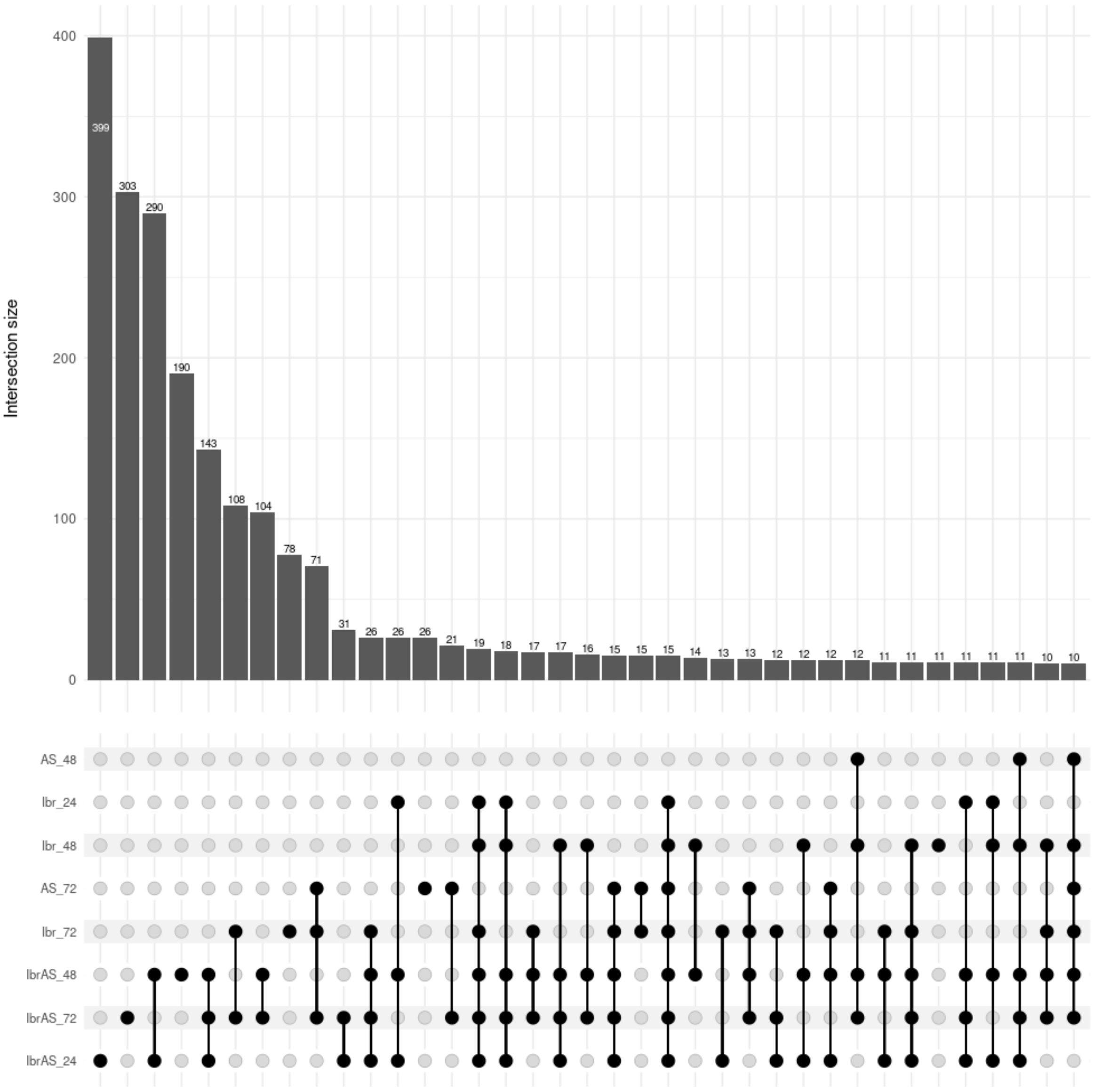


**Supplementary Figure 3. Upset plot of intersecting DEGs.** The combined treatment group induced the highest number of DEGs and shared the highest number of DEGs amongst the other treatment groups.

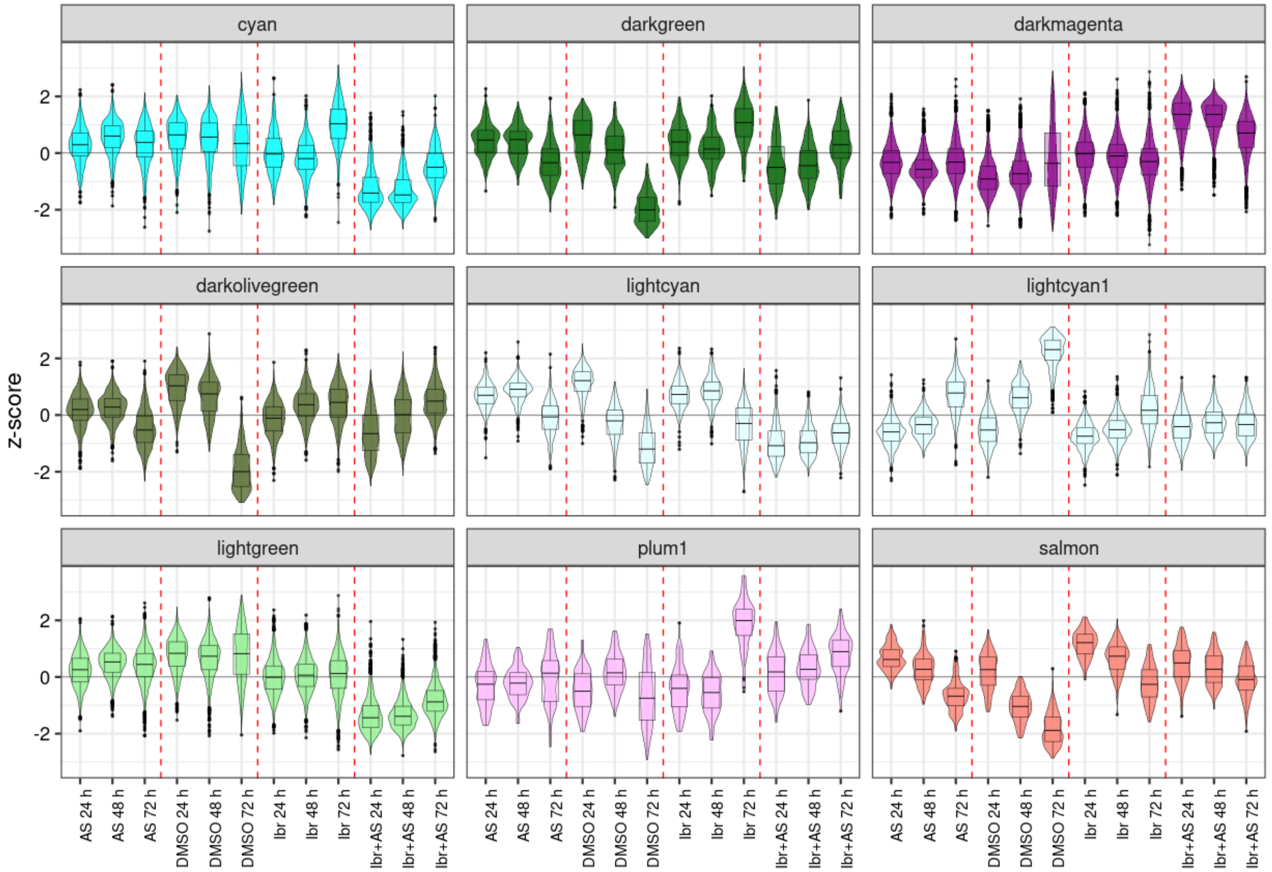


**Supplementary Figure 4. Expression profile z-scores of modules identified by WGCNA.** Gene expression tended to be lower in the *cyan*, *lightcyan* and *lightgreen* modules and higher in the *darkmagenta* module upon the combined Ibr+AS treatment. Furthermore, z-scores of *lightcyan1* and *salmon* modules increased and decreased respectively, with increasing incubation times.

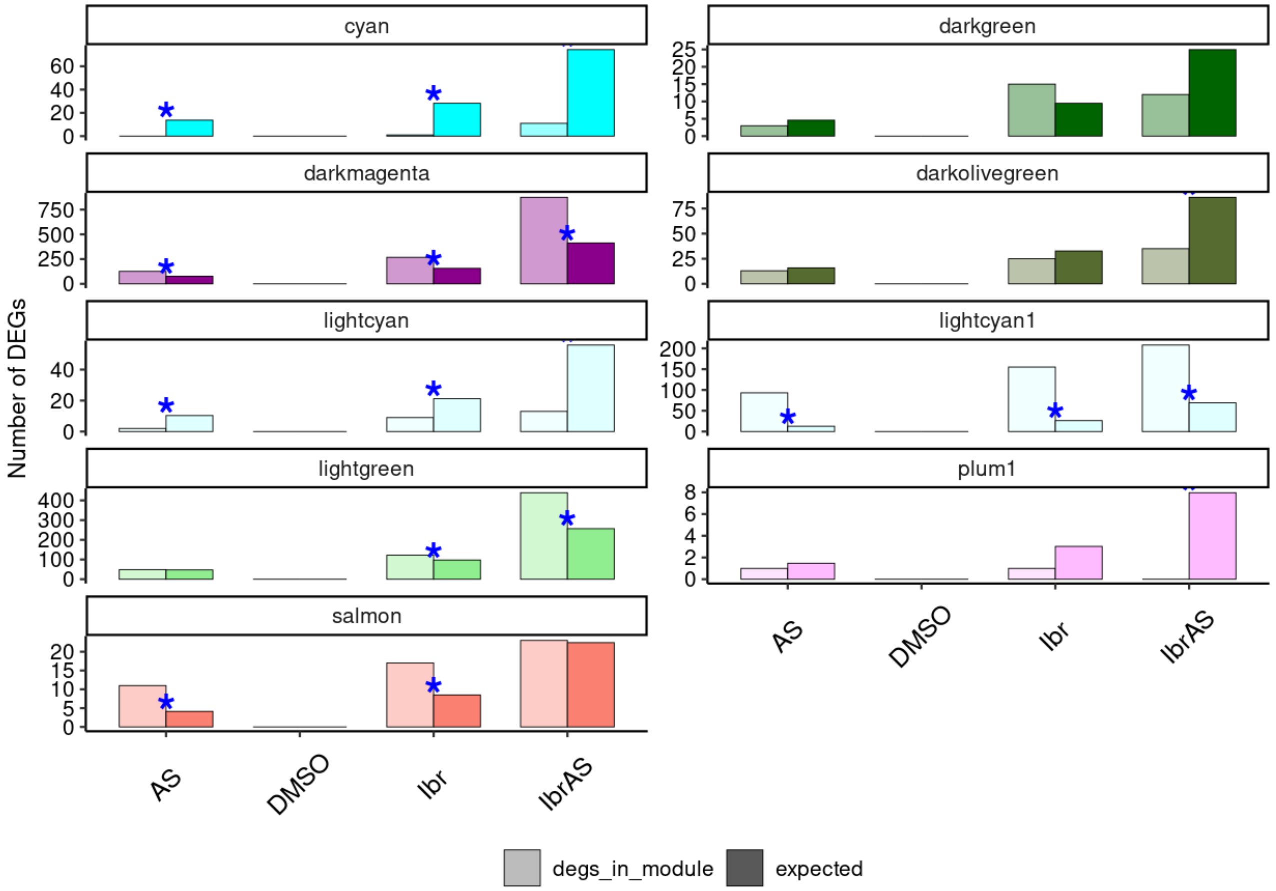


**Supplementary Figure 5. Bar Plot of expected vs. observed differentially expressed genes in each module and treatment group.** Significant Benjamini-Hochberg-adjusted *P*-values are indicated by blue stars and were computed with Fisher's exact test.
